# Open Clinical Trial Data for All? A View from Regulators

**DOI:** 10.1371/journal.pmed.1001202

**Published:** 2012-04-10

**Authors:** Hans-Georg Eichler, Eric Abadie, Alasdair Breckenridge, Hubert Leufkens, Guido Rasi

**Affiliations:** 1 European Medicines Agency (EMA), London, United Kingdom; 2 Agence Française de Sécurité Sanitaire des Produits de Santé (AFSSAPS) Saint-Denis, France; 3 Medicines and Healthcare products Regulatory Agency (MHRA), London, United Kingdom; 4 Medicines Evaluation Board (CBG-MEB), Den Haag, The Netherlands

## Abstract

Hans-Georg Eichler from the European Medicines Agency and colleagues provide a view from regulators on access to clinical trial data.

**Linked Policy Forum**

This Perspective discusses the following new Policy Forum published in *PLoS Medicine*:

Doshi P, Jefferson T, Del Mar C (2012) The Imperative to Share Clinical Study Reports: Recommendations from the Tamiflu Experience. PLoS Med 9(4): e1001201. doi:10.1371/journal.pmed.1001201

Peter Doshi and colleagues describe their experience trying and failing to access clinical study reports from the manufacturer of Tamiflu and challenge industry to defend their current position of RCT data secrecy.

In this issue of *PLoS Medicine*, Doshi and colleagues argue that the full clinical trial reports of authorized drugs should be made publicly available to enable independent re-analysis of drugs' benefits and risks [1]. We offer comments on their call for openness from a European Union drug regulatory perspective.

For the purpose of this discussion, we consider “clinical study reports” to comprise not just the protocol, summary tables, and figures of (mostly) randomized controlled trials (RCTs), but the full “raw” data set, including data at the patient level [2]. We limit discussion to data on drugs for which the regulatory benefit-risk assessment has been completed.

## Why Trial Data *Should* Be Open for All

First and foremost, we agree with Doshi et al. that clinical trial data should not be considered commercial confidential information; most patients enrolling in clinical trials do so with an assumption of contributing to medical knowledge, and “non-disclosure of complete trial results undermines the philanthropy” [1].

The potential benefits for public health of independent (re-)analysis of data are not disputed and, in an open society, trial sponsors and regulators do not have a monopoly on analyzing and assessing drug trial results. Yet, the different responsibilities of regulators and independent analysts have to be acknowledged. Regulators, unlike academicians, are legally obliged to take timely decisions on the availability of drugs for patients, even under conditions of uncertainty.

Going beyond the merits of independent meta-analysis, we foresee other, potentially more important benefits from public disclosure of raw trial data. For example, RCT datasets enabled the development of predictive models for patient selection to appropriate treatments [3],[4]. Taking this notion a step further, we envisage machine learning systems that will allow clinicians to match a patient's electronic health record directly to RCT and observational study data sets for better, individualized therapeutic decisions (L. Perez-Breva, personal communication).

Large, information-rich datasets are needed to support the computer science and artificial intelligence research required to develop and test these applications. Developing such tools is usually not a priority for, and often beyond the capabilities and resources of, even the largest pharmaceutical companies. These endeavors might best thrive in an environment that invites research from beyond the current stakeholders in health [5]. Making rich datasets available for research is a means to open health research.

## Why Trial Data *Should Not* Be Open for All

There are indeed many good arguments for unrestricted and easy access to full RCT data. Yet, simply uploading all trial data on a website would entail its own problems.

First among those is the issue of personal data protection or *patient* confidentiality, a concept that is very different from *commercial* confidentiality. There is a small risk that personal data could inadvertently be publicized. There is also a small risk that an individual patient could be identified from an anonymized dataset, for example, from trials in ultra-rare diseases. Achieving an adequate standard of personal data protection is not an insurmountable obstacle, though, and proposals for best practice for publishing raw data are available [2]. However, implementation is not straightforward, standards will need to be agreed upon up front, and data redaction may in a few cases be resource intensive.

Our second caveat is likely more contentious. We do not dispute that financial conflicts of interests (CoIs) may render analyses and conclusions “vulnerable to distortion” [1]. However, surrounding the ongoing debate over sponsor-independent analyses is an implicit assumption that “analysis by independent groups” is somehow free from CoIs. We beg to differ. Personal advancement in academia, confirmation of previously defended positions, or simply raising one's own visibility within the scientific community may be powerful motivators. In a publish-or-perish environment, would the finding of an important adverse or favorable drug effect at the *p*<0.05-level be more helpful to a researcher than *not* finding any new effects? Will society always be guaranteed that a finding that is reported as “confirmatory” was not the result of multiple exploratory re-runs of a dataset? We submit that analyses by sponsor-independent scientists are not generated in a CoI-free zone and, more often than not, ego trumps money. Independent analyses may therefore also be “vulnerable to distortion”. We are concerned that unrestricted availability of full datasets may in some cases facilitate the publication of papers containing misleading results, which in turn lead to urgent calls for regulatory action. In a worst case, this would give rise to unfounded health scares with negative public health consequences such as patients refusing vaccinations or discontinuing drug treatment [6],[7].

Aside from CoIs, independent analysis per se is no guarantee of high quality. The regulatory community has been confronted with meta-analyses that were later contradicted by additional evidence [8] or found to be flawed [9]. We argue that independent analyses warrant a similar level of scrutiny as sponsor-conducted analyses do.

Finally, re-analysis of trial data could be misused for competitive purposes.

## The Way Forward?

We consider it neither desirable nor realistic to maintain the status quo of limited availability of regulatory trials data. What is needed is a three-pronged approach:

1. Develop and agree upon adequate standards for protection of personal data when publicizing RCT datasets. Most stakeholders will likely agree that adequate standards of data protection are a *sine qua non*, so the issue should be primarily of a technical and legal nature. We emphasize *adequate* standards because excessive demands and unrealistically high standards may in effect become an “anti-commons” and frustrate important public health gains.
2. Ensure general adoption of established quality standards of meta-analyses and other types of (confirmatory) data re-analysis that may warrant regulatory action.
3. Establish rules of engagement: In the area of observational studies based on health care databases, the European Network of Centres for Pharmacoepidemiology and Pharmacovigilance (ENCePP) has recently published guidance for raw data sharing; these rules of engagement follow the principle of maximum transparency whilst respecting the need to guarantee data privacy and to avert the potential for misuse [10]. Others have come up with broadly similar proposals [11]. Conceivably, analogous principles (e.g., data sharing only after receipt of a full analysis plan) could be applied to regulatory RCT data [1].

Moreover, we take it as self-evident that the same standard of openness should apply to all (drug) trial data, whether sponsored by industry, investigator-initiated, or sponsored by public grant-giving bodies. Likewise, the same standard of third party scrutiny should be applicable to all secondary data analyses. Regulatory inspections of data and analyses carried out by commercial sponsors are routine. Would all sponsor-independent researchers allow the same level of inspections applied to their analyses?

We welcome debate on these issues, and remain confident that satisfactory solutions can be found to make complete trial data available in a way that will be in the best interest of public health.

## Abbreviations

CoI: conflict of interest
RCT: randomized controlled trial
